# Evidence for a GPR18 Role in Diurnal Regulation of Intraocular Pressure

**DOI:** 10.1167/iovs.16-19437

**Published:** 2016-11

**Authors:** Sally Miller, Emma Leishman, Olivia Oehler, Laura Daily, Natalia Murataeva, Jim Wager-Miller, Heather Bradshaw, Alex Straiker

**Affiliations:** The Gill Center for Biomolecular Science and the Department of Psychological and Brain Sciences, Indiana University, Bloomington, Indiana, United States

**Keywords:** intraocular pressure, glaucoma, diurnal, circadian, eye, cannabinoid, anandamide, arachidonoyl ethanolamide, NAPE-PLD, FAAH

## Abstract

**Purpose:**

The diurnal cycling of intraocular pressure (IOP) was first described in humans more than a century ago. This cycling is preserved in other species. The physiologic underpinning of this diurnal variation in IOP remains a mystery, even though elevated pressure is indicated in most forms of glaucoma, a common cause of blindness. Once identified, the system that underlies diurnal variation would represent a natural target for therapeutic intervention.

**Methods:**

Using normotensive mice, we measured the regulation of ocular lipid species by the enzymes fatty acid amide hydrolase (FAAH) and *N*-arachidonoyl phosphatidylethanolamine phospholipase (NAPE-PLD), mRNA expression of these enzymes, and their functional role in diurnal regulation of IOP.

**Results:**

We now report that NAPE-PLD and FAAH mice do not exhibit a diurnal cycling of IOP. These enzymes produce and break down acylethanolamines, including the endogenous cannabinoid anandamide. The diurnal lipid profile in mice shows that levels of most *N*-acyl ethanolamines and, intriguingly, *N*-arachidonoyl glycine (NAGly), decline at night: NAGly is a metabolite of arachidonoyl ethanolamine and a potent agonist at GPR18 that lowers intraocular pressure. The GPR18 blocker O1918 raises IOP during the day when pressure is low, but not at night. Quantitative PCR analysis shows that FAAH mRNA levels rise with pressure, suggesting that FAAH mediates the changes in pressure.

**Conclusions:**

Our results support FAAH-dependent NAGly action at GPR18 as the physiologic basis of the diurnal variation of intraocular pressure in mice.

The diurnal regulation of intraocular pressure (IOP) has been known for more than a century,^1,2^ yet the physiologic underpinnings of this regulation remain a mystery. In humans, IOP rises during the day and falls at night. There is general agreement that diurnal variation occurs in humans, with most studies finding pressure rises in early to late morning,^3^ although there is disagreement regarding the precise timing and amplitude of the variation. Various aspects of the rhythm have been studied,^4^ but the molecular and cellular basis for this variation (the means by which the circadian clock genes and proteins regulate pressure) is unknown. This is despite the fact that elevated IOP is a major risk factor for primary open-angle glaucoma, and the standard therapeutic approach for glaucoma remains the lowering of IOP.^5^ Identification of the underpinnings of the diurnal cycling of IOP therefore represents a potential therapeutic point of intervention for glaucoma. The rhythm is present in other animals such as the rabbit^6^ and the mouse.^7^ Using mouse models, it has been determined that circadian rhythm clock genes are implicated in the regulation of this rhythm because *Cry1* and *Cry2* mutants lack this diurnal variation^8^ and various clock-related genes change their expression in nonpigmented epithelium of the mouse cycle in tandem with IOP.^7^

We previously tested components of the cannabinoid signaling system, some of which regulate IOP, for a potential role in this diurnal oscillation of ocular pressure, finding that knockout mice for the cannabinoid and cannabinoid-related receptors CB_1_, CB_2_, and GPR55 retain their diurnal IOP rhythm.^9,10^ More recently, we reported that activation of GPR18 also lowers IOP and that the receptors are present in multiple tissues in the anterior eye.^10^ GPR18 is a lipid receptor that is activated by a metabolite of endocannabinoids as described in greater detail below. In the course of a lipid analysis of cannabinoid-related enzyme knockout mice, we noted a curious diurnal variation in several key lipids, prompting the current study.

Endocannabinoids, most notably 2-arachidonoyl glycerol (2-AG) and *N*-arachidonoyl ethanolamine (AEA, anandamide), are endogenous lipid ligands for the cannabinoid receptors CB_1_ and CB_2_.^11–14^ These lipids have several notable characteristics, one being that they are produced enzymatically and on demand,^15^ and much effort has gone into identification of the responsible enzymes both on the production and metabolism side. Another important feature of these endocannabinoids is that they have multiple congeners that differ by the length and saturation of their carbon backbone. The 2-AG–related lipid species 2-oleoyl glycerol (2-OG), 2-palmitoyl glycerol (2-PG), and 2-linolenoyl glycerol (2-LG) are all present in the CNS at comparable quantities.^16,17^ Some of them may have their own roles as ligands: 2-OG has been proposed as an endogenous ligand for GPR119.^18^ Because a given enzyme may metabolize an entire class of lipids (e.g., monoacylglycerol lipase (MAGL) metabolizes not only 2-AG but also 2-PG, 2-OG, and 2-LG,^19^ the picture of signaling is muddied. In the case of 2-AG and AEA, the congeners have been proposed to play the role of “entourage” compounds, competing for breakdown and thereby enhancing the signaling of the chief compound,^20^ although this is still a subject of debate.^21^ Because of this complexity, it is important to study not only the disposition of the lipids of interest but also of the related lipid species. Enzymatic processes are best conceived of as dynamic flows; blockade or elimination of key enzymes can block these flows the way a dam might block a river. The accumulation of molecules—the reservoir that builds behind the dam, to extend the metaphor—may offer insights into the nature of those flows. We therefore examined the changes that result from the blockade of two enzymes: fatty acid amide hydrolase (FAAH) and *N*-acyl phosphatidylethanolamine-specific phospholipase D (NAPE-PLD). These enzymes have been implicated in the metabolism of anandamide, which has been detected in various ocular tissues.^22,23^ The role of FAAH in breaking down anandamide was established relatively early,^24^ and anandamide metabolism, presumed to occur via FAAH activity, has been studied in the eye.^25,26^ Importantly in our case, FAAH and anandamide are implicated in the production of *N*-arachidonoyl glycine (NAGly), a candidate GPR18 agonist.^27,28^ NAPE-PLD was proposed early on as the enzymatic source of anandamide,^29,30^ but the first knockout study was discouraging, setting off a search for other candidate pathways.^31^ Recently this question has been revisited and further support has been lent to a central role of NAPE-PLD in the production of anandamide in particular but also other acylethanolamines.^32^

We now present an examination of the regulation of ocular lipids by these enzymes, how these lipids and enzymes change under diurnal conditions, and a functional consequence of this regulation.

## Methods

### Animals

Experiments were conducted at the Indiana University campus. All mice used for IOP experiments were handled according to the guidelines of the Indiana University animal care committee. Mice (male, age 3–8 months) were kept on a 12-hour (0600–1800 hours) standard light dark cycle (SLC) or a reverse light cycle (RLC) and fed ad libitum. Male C57BL/6J (C57) mice were obtained from Charles River Laboratories International, Inc. (Wilmington, MA, USA) or were kindly provided by Ken Mackie (Indiana University, Bloomington, IN, USA). Mice were allowed to acclimatize to the animal care facility for at least a week prior to their use in experiments. A total of 133 animals were used in these experiments. FAAH^−/−^, GPR119^−/−^, and NAPE-PLD^−/−^ mice were kindly provided by Ken Mackie. The knockouts are all global knockouts and do not exhibit obvious phenotypic abnormalities. FAAH^−/−^ animals were originally developed in the laboratory of Ben Cravatt (Scripps Research Institute, La Jolla, CA, USA); NAPE-PLD^−/−^ mice were developed in the laboratory of Richard Palmiter (University of Washington, Seattle, WA, USA); and GPR119^−/−^ mice were originally obtained from the Mutant Mouse Regional Resource Center at the University of California (Davis, CA, USA).

### Light Cycles

For most experiments including lipid measurements and diurnal IOP measurements, animals were maintained on a standard light cycle and then tested at noon or midnight. The exception was the O1918 experiment, where the animals were treated at noon, having been maintained on an SLC or RLC (to mimic night-time conditions).

### Intraocular Pressure Measurements

Intraocular pressure was measured in mice by rebound tonometry, using a Tonolab (Icare Finland Oy, Helsinki, Finland). This instrument uses a light plastic-tipped probe to briefly make contact with the cornea; after the probe encounters the eye, the instrument measures the speed at which the probe rebounds to calculate IOP.

To obtain reproducible IOP measurements, mice were anesthetized with isoflurane (3% induction). The anesthetized mouse was then placed on a platform in a prone position, where anesthesia was maintained with 2% isoflurane. Baseline IOP measurements are taken in both eyes. A measurement consisted of the average value of six readings. A minimum of three measurements were taken for each time point. One eye was then treated with drug (dissolved in Tocrisolve, a soya-based solvent,^33^ 5-μL final volume applied topically; Tocris, Ellisville, MO, USA) while the other eye was treated with vehicle. The animal was then allowed to recover (recovery from isoflurane is rapid, typically a few minutes). After an hour, the animal was again anesthetized as above. Intraocular pressure was then measured in the drug-treated and vehicle-treated contralateral eye. Intraocular pressure measurements were analyzed by paired *t*-tests comparing the drug-treated eyes to vehicle-treated eyes or same-animal diurnal values at noon or midnight.

### Lipid Extraction, HPLC Tandem Mass Spectroscopy, and Quantitative PCR

For details, please see the Supplementary Methods.

### Drugs

O-1918 was obtained from Cayman Chemical (Ann Arbor, MI, USA). Tocrisolve was obtained from Tocris. Topically applied drugs were prepared by dilution in Tocrisolve.

## Results

### Ocular *N*-Acyl Ethanolamines Are Dramatically Increased in FAAH Knockout Mice

As noted above, FAAH is strongly implicated in the metabolism of *N*-acyl ethanolamines (NAEs), among them the CB_1_ agonist anandamide.^24^ Although most attention has focused on the canonical cannabinoids AEA and 2-AG, the body produces a range of related lipids, often via the same enzymes. What function—if any—these might have is still largely an open question. We therefore examined a panel of ∼80 lipids, the full list of which is found in Supplementary Table S1. Twenty-four members of this panel included the oleoyl-, arachidonoyl-, palmitoyl-, stearoyl-, linoleoyl-, and docosahexaenoyl-based NAEs, *N*-acyl GABAs, *N*-acyl glycines, and *N*-acyl serines. The list additionally included linoleic and arachidonic free fatty acids, three acyl-glycerols (including the chief endocannabinoid 2-AG, 2-OG, and 2-LG), the prostaglandin metabolites prostaglandin E_2_ (PGE_2_) and prostaglandin E_2_-glycerol (PGE_2_G), and *N*-arachidonoyl taurine.

**Figure 1:**
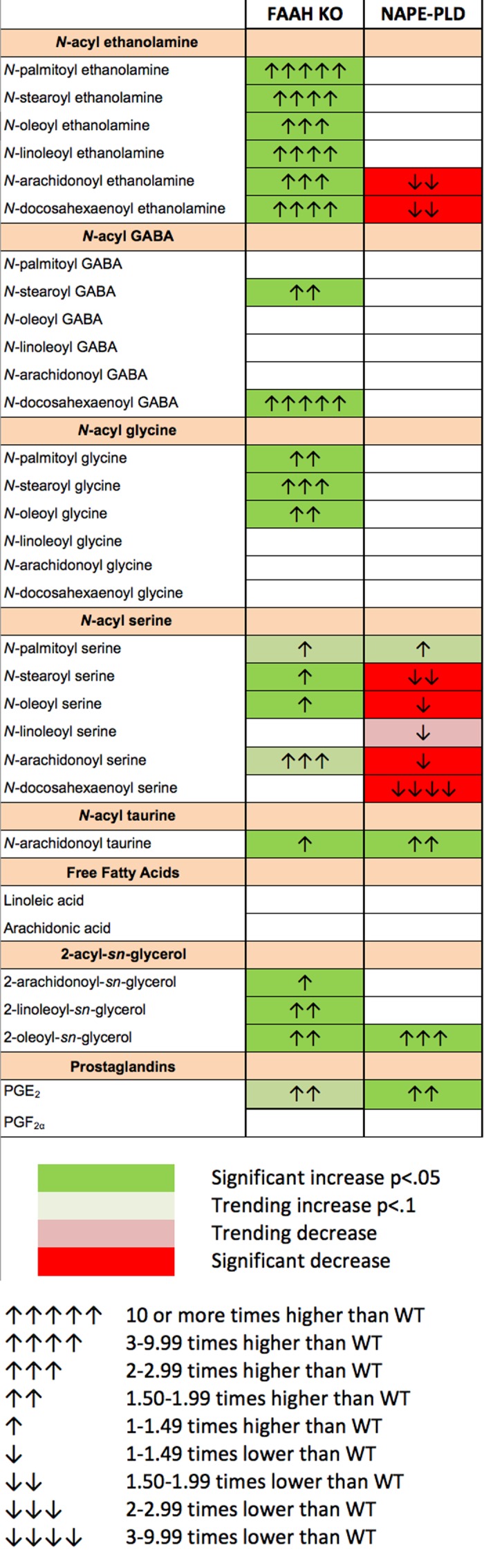
Changes in cannabinoid-related lipid species in the eye of FAAH and NAPE-PLD knockout mice. Changes in lipid species relative to WT. Consistent with expectations, FAAH eyes saw large increases in the NAEs, but also in other lipid species including several *N*-acyl glycines and the 2-acyl-sn-glycerols, among them 2-AG. In contrast, NAPE-PLD eyes changed more modestly, mostly declining.

Figure 1 shows changes in lipid levels in FAAH knockout mice relative to age- and sex-matched wild-type (WT) mice, omitting those classes that did not exhibit a change. Consistent with the expected role for FAAH, we found levels of all NAEs tested to be higher in eyes of FAAH knockout mice relative to WT mice. All were at least doubled relative to WT, and one of these, *N*-palmitoyl ethanolamine, was found to be more than 10 times higher in the knockout. Interestingly, the acyl glycerols, which include the endocannabinoid 2-AG, 2-LG, and 2-OG, were also elevated as a group, although not as greatly as the NAEs. The majority of the *N*-acyl glycine lipids increased in the FAAH KO, and one species of *N*-acyl GABA, *N*-docosahexaeonyl GABA, was only detectable in the FAAH KO, suggesting that the levels in the WT were dramatically lower.

### Some *N*-Acyl Ethanolamines and *N*-Acyl Serines Decline in NAPE-PLD Knockout Mice

On the production side, as previously noted, NAPE-PLD has recently been re-implicated as an enzymatic source of NAEs. We therefore conducted a similar experiment using NAPE-PLD knockout mice relative to WT controls, the results of which are summarized in Figure 1. Interestingly, only a few lipid species were found to be altered. As hypothesized, AEA levels declined in the NAPE-PLD knockout. Levels of *N*-docosahexaenoyl ethanolamine declined, as did most of the *N*-acyl serines. The only lipid species that rose in NAPE-PLD knockouts were *N*-arachidonoyl taurine, 2-OG, and, interestingly, prostaglandin E2.

### *N*-Acyl Ethanolamines and NAGly Vary Diurnally in the Eye of the WT Mouse

**Figure 2:**
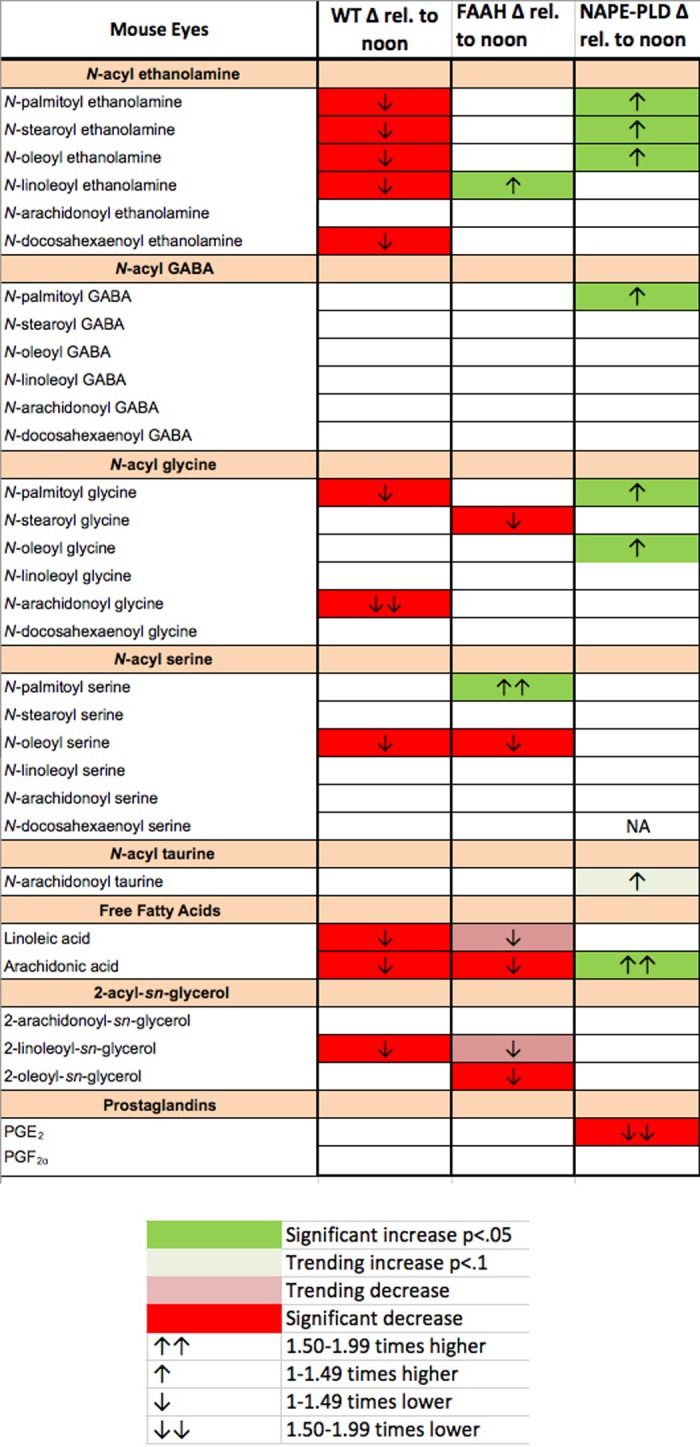
Diurnal variation in cannabinoid-related lipid species in WT, FAAH^−/−^, and NAPE-PLD^−/−^ eyes. Several cannabinoid-related lipid species declined at midnight relative to noon in mouse WT eyes including most NAEs, with the strongest decline in NAGly. The FAAH and NAPE-PLD knockout animals did not see the same changes.

To learn whether any cannabinoid-related lipids exhibited diurnal variation, we tested the same lipid panel in WT mouse eyes harvested at noon or midnight. The results are summarized in the left-most column of Figure 2. Interestingly there is a consistent pattern of decline in NAEs at night. With the notable exception of *N*-arachidonoyl ethanolamine (aka anandamide), all NAEs declined in eyes harvested at midnight relative to noon. Notably, the most pronounced change was seen for NAGly, a metabolite of the aforementioned anandamide^27^ and an agonist at GPR18.^34^ Some other lipids including arachidonic acid declined at night but the most striking pattern involves the NAEs and NAGly.

Consequently, we tested whether the diurnal pattern was maintained in knockout mice for FAAH, which breaks down NAEs and is implicated in NAGly synthesis,^27^ and in knockouts for NAPE-PLD, implicated in their production as noted previously. We found that the diurnal variation in NAEs was absent in FAAH knockouts and, if anything, reversed for NAPE-PLD knockouts. NAGly variation was also absent in both knockouts.

### Diurnal Variation of IOP Is Absent in NAPE-PLD and FAAH Knockout Mice

**Figure 3:**
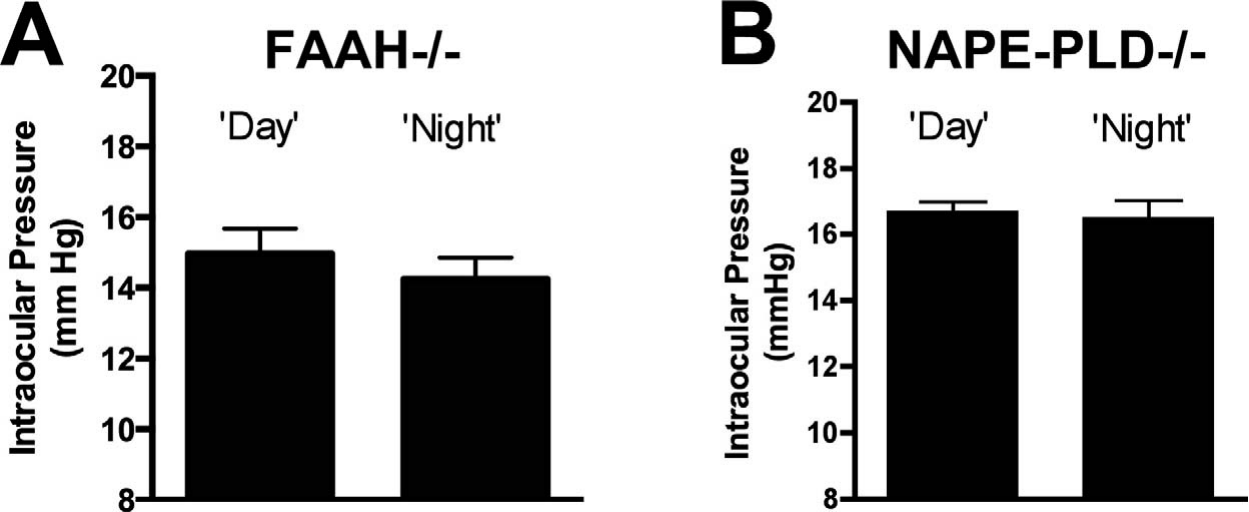
The FAAH and NAPE-PLD knockout mice do not exhibit diurnal variation in intraocular pressure. (**A**) Intraocular pressure readings in FAAH^−/−^ mice taken during at noon (day) or midnight (night). (**B**) Diurnal IOP readings for NAPE-PLD^−/−^ mice. NS, *P* < 0.05, paired *t*-test.

In the mouse, IOP rises reliably at night.^7^ Because *N*-acylethanolamines and NAGly varied diurnally in WT mice in a manner consistent with downward regulation of IOP during the day when their levels are highest, we tested whether diurnal variation of IOP was maintained in FAAH and NAPE-PLD knockout mice. As shown in Figure 3, diurnal variation in IOP was absent in both FAAH (Fig. 3A; IOP at noon [mm Hg]: 15.0 ± 0.7; at midnight: 14.3 ± 0.6; *n* = 22; *P* > 0.05 paired *t*-test) and NAPE-PLD (Fig. 3B; IOP at noon [mm Hg]; 16.7 ± 0.3; at midnight: 16.5 ± 0.5, *n* = 12; *P* > 0.05, paired *t*-test) knockout mice.

Taken together with our lipid analysis, this implicates several potential candidates in diurnal regulation of IOP: the NAEs (minus anandamide), *N*-palmitoyl glycine (PalGly), and NAGly. The other compounds all exhibited a change in either FAAH or NAPE-PLD knockouts. We previously reported that this diurnal variation in IOP persists in CB_1_, CB_2_, and GPR55 knockout animals, indicating that these receptors are not involved.^9,10^ Several of the candidate molecules have been linked to GPR119 or GPR18: GPR119 may be a target for *N*-oleoyl ethanolamine (OEA) and *N*-palmitoyl ethanolamine (PEA),^35^ whereas GPR18 has been proposed as the endogenous partner for NAGly. Notably, activation of GPR18 by NAGly lowers IOP in the eye.^10^ We tested IOP in GPR119 knockout mice, finding that the diurnal rhythm was intact in these mice (data not shown; daytime IOP [mm Hg]: 14.6 ± 0.5; nighttime: 19.2 ± 1.2, *n* = 8, *P* < 0.01, paired *t*-test). We next turned to GPR18.

### The GPR18 Antagonist O-1918 Raises IOP When Pressure Is Low

**Figure 4:**
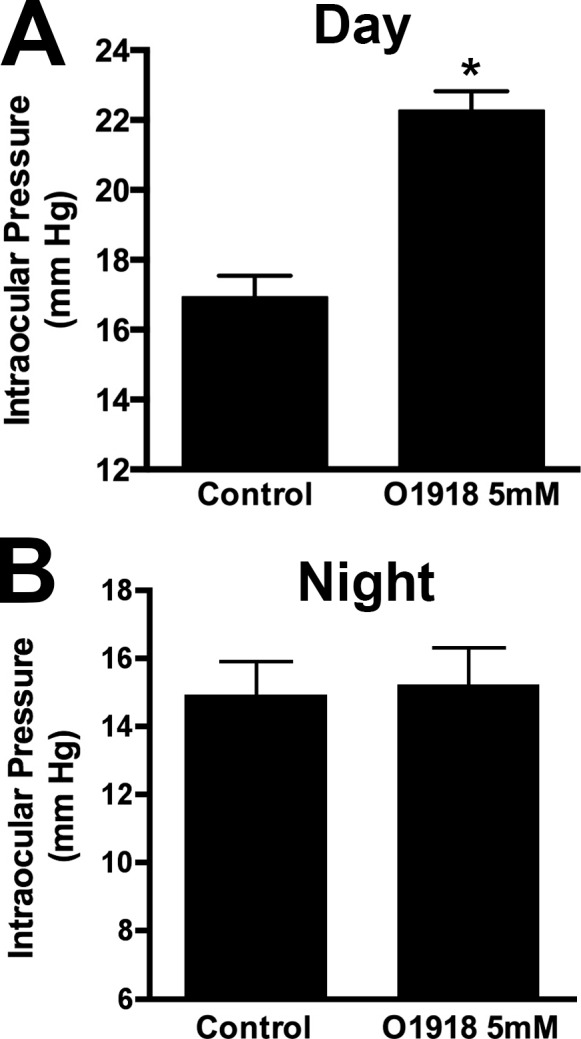
The GPR18 antagonist O-1918 raises IOP during the day but not at night. (**A**) Intraocular pressure in O-1918–treated versus control eye in midday treatments. (**B**) Same treatment in separate group of animals under reverse light cycle, equivalent to midnight. *P < 0.05, paired *t*-test.

As noted above, we previously showed that GPR18 agonists lower IOP in a normotensive mouse model. The GPR18 agonist NAGly is also the lipid that exhibits the most pronounced drop at night. If NAGly mediates the diurnal variation in IOP, then one would predict that a GPR18 antagonist applied to the eye of a mouse during the day, when pressure is low, would actually raise IOP. As shown in Figure 4, pressure was found to increase during the day but not at night in response to treatment with the GPR18 antagonist O-1918 (baseline IOP at noon [mm Hg]: 16.9 ± 0.6: O-1918 [5 mM]: 22.3 ± 0.5; *n* = 8, *P* < 0.05, paired *t*-test; baseline [midnight equivalent]: 14.9 ± 1.0; O-1918 [5 mM]: 15.2 ± 1.1, *n* = 16, not significant, paired *t*-test).

### Messenger RNA Levels for FAAH but Not GPR18 and NAPE-PLD Vary Diurnally

**Figure 5:**
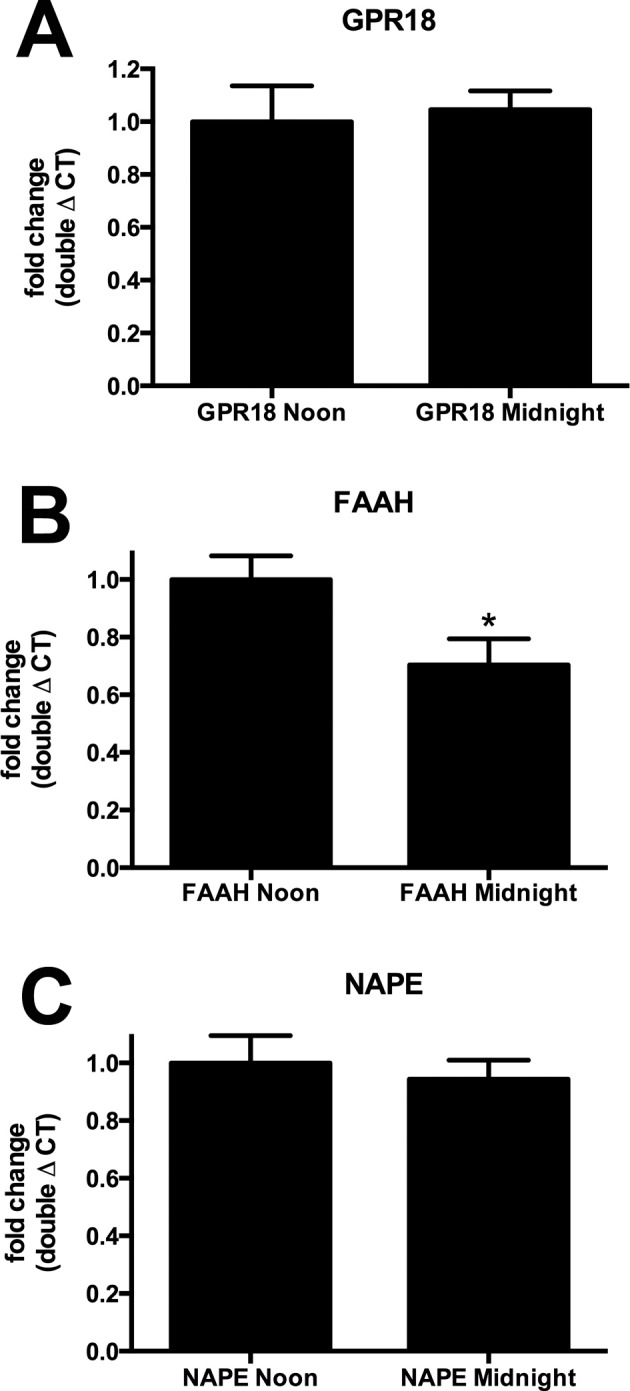
Fatty acid amide hydrolase mRNA levels decline at midnight relative to noon. (**A**) Messenger RNA levels for GPR18 were unchanged at midnight relative to noon in mouse eyes using quantitative PCR analysis. (**B**) Fatty acid amide hydrolase levels were lower at midnight. (**C**) *N*-arachidonoyl phosphatidylethanolamine phospholipase did not vary diurnally. *P < 0.05 unpaired *t*-test, *n* = 6.

Our results implicate GPR18, FAAH, and NAPE-PLD in the diurnal regulation of IOP, but do not address the point of regulation. To raise levels of NAGly and thereby lower pressure during the day, presumably either NAPE-PLD levels and/or activity are enhanced or FAAH levels and/or activity are altered. The situation for FAAH is more complicated because there are two chief hypothetical pathways for NAGly synthesis.^27^ One involves FAAH-mediated synthesis from arachidonic acid, whereas the other involves a two-step process via alcohol dehydrogenase. In the former case, one would predict that activity and/or levels of FAAH would need to rise to increase levels of NAGly. In the latter case, FAAH could enhance NAGly levels by impeding the breakdown of anandamide, thereby leaving more precursor for the conversion to NAGly. Thus, in either metabolic pathway, if FAAH is the point of regulation, one would expect an increase in FAAH activity and/or expression to be associated with elevated NAGly. To explore this question, we examined the levels of mRNA for NAPE-PLD and FAAH at noon and midnight. Alhough our lipid analyses implicate the enzymes, it is possible that GPR18 levels also change, and we therefore included an analysis of GPR18 in our study. We find that GPR18 and NAPE-PLD levels do not vary (Fig. 5A; GPR18 midnight [fold change normalized to noon ± SEM]: 1.05 ± 0.07; *n* = 6; Fig. 5C; NAPE-PLD midnight: 0.94 ± 0.07; *P* > 0.05, unpaired *t*-test noon versus midnight for respective genotypes), but that FAAH levels are relatively higher during the day (Fig. 5B; FAAH midnight [fold change normalized to noon ± SEM]: 0.70 ± 0.09; *n* = 6, *P* < 0.05, unpaired *t*-test noon versus midnight).

## Discussion

Our chief findings are that knockout mice for the enzymes implicated the production and breakdown of cannabinoid-related acylethanolamines, FAAH and NAPE-PLD, do not exhibit a diurnal variation in their IOP. We find that variation is intact in GPR119 knockout mice and that a GPR18 antagonist raises IOP when it is low but not when it is high. The point of regulation appears to involve differential FAAH expression because the IOP rhythm is absent in both FAAH and NAPE-PLD knockouts but only FAAH levels rise in concert with NAGly. Taken together, the data suggest a model whereby NAPE-PLD produces *N*-arachidonoyl ethanolamine (anandamide) that is converted by FAAH to NAGly, which then lowers IOP via activation of GPR18. These results reinforce GPR18 as a potential therapeutic target for the treatment of glaucoma. A GPR18 agonist may serve to reduce pressures at a time during the diurnal cycle when they are highest and presumably most likely to damage the eye.

There are several hypotheses regarding the role of FAAH in NAGly synthesis.^27^ FAAH is a demonstrated metabolizing enzyme for NAEs and anandamide has been shown to serve as a precursor for NAGly, but FAAH may serve a dual role by also synthesizing NAGly from arachidonic acid and glycine.^27^ Alternatively, NAGly may be formed by a two-step process involving alcohol dehydrogenase.^27^ In the former case, one would predict that FAAH blockade would result in elevated anandamide but not NAGly relative to WT because the accumulated anandamide would not be further metabolized to NAGly. Our combination of lipid and protein analyses support this hypothesis. Our quantitative analysis of mRNA is also consistent with FAAH as the point of regulation for diurnal variation of IOP because FAAH levels rise in concert with NAGly.

In terms of therapeutic applications, the targeting of FAAH would initially appear to be desirable because it enhances endogenous levels of anandamide, which can lower IOP for several hours in animal models.^36–38^ Anandamide has additionally been shown to mediate an increase in outflow of aqueous humor using an organ culture model, in a CB_1_- and CB_2_-dependent manner,^39^ although we do not see a role for CB_2_ in the mouse.^10^ However, AEA effects on IOP in rabbit were insensitive to the CB_1_ receptor antagonist SR141716^40^ but were blocked by an inhibitor of prostaglandin synthesis,^37^ suggesting that the actions of AEA are CB_1_-independent and instead due to activity of prostaglandins. AEA metabolism has been detected in several porcine ocular tissues.^38^ Blockade of FAAH was found to have salutary effects on retina in the wake of high IOP-induced ischemia.^41^ FAAH blockade therefore has therapeutic appeal but presumably comes at the cost of preventing the diurnal drop in IOP that relies on an active FAAH.

Differential expression of FAAH is implicated as the underpinning of changes in NAGly levels and by extension IOP. However, what regulates FAAH expression and how is this linked to a circadian clock? It is possible that FAAH gene expression is regulated by clock-related genes that were recently found to cycle in tandem with the IOP rhythm, such as *Per1* and *Per2*.^7^ *Cry1* and *Cry2* are also candidates, particularly because mutants for these receptors also do not exhibit a diurnal IOP rhythm.^8^ There may, however, be intermediaries between these systems and FAAH expression. And where is FAAH expressed to regulate this production? We recently showed that FAAH mRNA is expressed in cornea, trabecular meshwork, and retina of the cow, the three tissues tested.^42^

Our results also point to other potentially interesting roles for FAAH and NAPE-PLD in metabolism of cannabinoid-related lipids, the significance of which are unknown at this time. For instance, what is the significance of the >10-fold changes in docosahexaenoyl serine levels in NAPE-PLD knockout mice and docosahexaenoyl GABA in FAAH knockout mice? It is possible that some of the remaining lipids in this panel for which there is currently no known role will be found to partner with orphan GPCRs or other receptors.^43^ It is difficult to interpret the remaining changes in lipid levels seen in WT, NAPE-PLD, and FAAH knockout mice. Although statistically significant, some of these may be secondary or even tertiary effects as levels of precursors for other lipids are skewed in a dynamic system. The rise of arachidonic acid and decline of PGE_2_ are the most pronounced changes in NAPE-PLD knockouts. The rise in arachidonic acid may be an indication that arachidonoyl lipid species are important in the arachidonic acid synthesis cycle, with implications for prostaglandin synthesis. AEA has been reported to induce a biphasic effect on IOP (an initial rise followed by a prolonged drop),^36^ both being blocked by an inhibitor of prostaglandin synthesis.^37^ This role would contrast with the finding for MAGL in the brain; MAGL has been implicated in arachidonic acid especially in the CNS,^44^ and blockade of MAGL, by locking up arachidonic acid species in the form of 2-AG, can lead to dramatic decreases in prostaglandin levels.

In summary, our results implicate GPR18 as the receptor mediating the diurnal variation in IOP. We propose a model whereby FAAH expression varies diurnally in the mouse, resulting in enhanced production of the anandamide metabolite NAGly during the day, actively lowering pressure via GPR18. It is likely that this mechanism will be conserved in humans, although perhaps reversed because pressure is higher during the day, meaning that the GPR18 system would be active at night. However, it will be important as a first step to verify that a GPR18 signaling system is present in the human eye and that activation of this receptor lowers IOP. If the system is conserved, then harnessing of the inactive GPR18 system during the day in humans would represent a desirable therapeutic target.

## Supplementary Material

Supplement 1

Supplement 2
