# Supplementary material for: Evidence for a GPR18 Role in Diurnal Regulation of Intraocular Pressure
Source: Invest Ophthalmol Vis Sci. 2016 Nov;57(14):6419–26. doi: 10.1167/iovs.16-19437 (PMC5132083; doi:10.1167/iovs.16-19437)
Supplement: Supplement 1 [file iovs-57-13-48_s01.pdf]

***N*-acyl alanine**	**[M – H]$^-$**	**Fragment**
N-palmitoyl alanine	326.5	88.09
N-stearoyl alanine	354.55	88.09
N-oleoyl alanine	352.53	88.09
N-linoleoyl alanine	350.52	88.09
N-arachidonoyl alanine	374.5	88.09
N-docosahexaenoyl alanine	398.56	88.09

***N*-acyl ethanolamine**	**[M – H]$^-$**	**Fragment**
N-palmitoyl ethanolamine	300.29	62.1
N-stearoyl ethanolamine	328.3	62.1
N-oleoyl ethanolamine	326.3	62.1
N-linoleoyl ethanolamine	324.3	62.1
N-arachidonoyl ethanolamine	348.29	62.1
N-docosahexaenoyl ethanolamine	372.6	62.1

***N*-acyl GABA**	**[M – H]$^-$**	**Fragment**
N-palmitoyl GABA	340.54	102.1
N-stearoyl GABA	368.58	102.1
N-oleoyl GABA	366.57	102.1
N-linoleoyl GABA	364.54	102.1
N-arachidonoyl GABA	388.57	102.1
N-docosahexaenoyl GABA	412.59	102.1

***N*-acyl glycine**	**[M – H]$^-$**	**Fragment**
N-palmitoyl glycine	312.26	74.2
N-stearoyl glycine	340.3	74.2
N-oleoyl glycine	338.3	74.2
N-linoleoyl glycine	336.3	74.2
N-arachidonoyl glycine	360.3	74.2
N-docosahexaenoyl glycine	384.3	74.2

***N*-acyl leucine**	**[M – H]$^-$**	**Fragment**
N-palmitoyl leucine	368.58	130.1
N-stearoyl leucine	396.63	130.1
N-oleoyl leucine	394.61	130.1
N-linoleoyl leucine	392.6	130.1
N-docosahexaenoyl leucine	440.64	130.1

***N*-acyl methionine**	**[M – H]$^-$**	**Fragment**
N-palmitoyl methionine	386.62	148.2
N-stearoyl methionine	414.64	148.2
N-oleoyl methionine	412.65	148.2
N-linoleoyl methionine	410.64	148.2
N-arachidonoyl methionine	434.66	148.2
N-docosahexaenoyl methionine	458.68	148.2

***N*-acyl phenylalanine**	**[M – H]$^-$**	**Fragment**
N-palmitoyl phenylalanine	402.59	164.1
N-stearoyl phenylalanine	430.65	164.1
N-oleoyl phenylalanine	428.63	164.1
N-linoleoyl phenylalanine	426.61	164.1
N-arachidonoyl phenylalanine	450.64	164.1
N-docosahexaenoyl phenylalanine	474.66	164.1

***N*-acyl proline**	**[M – H]$^-$**	**Fragment**
N-palmitoyl proline	352.53	114.12
N-stearoyl proline	380.59	114.12
N-oleoyl proline	378.31	114.12
N-linoleoyl proline	376.56	114.12
N-arachidonoyl proline	400.58	114.12
N-docosahexaenoyl proline	424.6	114.12

***N*-acyl serine**	**[M – H]$^-$**	**Fragment**
N-palmitoyl serine	342.3	74
N-stearoyl serine	370.3	74
N-oleoyl serine	368.3	74
N-linoleoyl serine	366.27	74
N-arachidonoyl serine	390.3	74
N-docosahexaenoyl serine	414.3	74

***N*-acyl taurine**	**[M – H]$^-$**	**Fragment**
N-arachidonoyl taurine	410.6	124

***N*-acyl tryptophan**	**[M – H]$^-$**	**Fragment**
N-palmitoyl tryptophan	441.63	203.1
N-stearoyl tryptophan	469.68	203.1
N-oleoyl tryptophan	467.67	203.1
N-linoleoyl tryptophan	465.65	203.1
N-arachidonoyl tryptophan	489.67	203.1
N-docosahexaenoyl tryptophan	513.69	203.1

***N*-acyl tyrosine**	**[M – H]$^-$**	**Fragment**
N-palmitoyl tyrosine	418.59	180.18
N-stearoyl tyrosine	446.65	180.18
N-oleoyl tyrosine	444.63	180.18
N-linoleoyl tyrosine	442.61	180.18
N-arachidonoyl tyrosine	466	180.18
N-docosahexaenoyl tyrosine	490.66	180.18

***N*-acyl valine**	**[M – H]$^-$**	**Fragment**
N-palmitoyl valine	354.31	116.31
N-stearoyl valine	382.6	116.14
N-oleoyl valine	380.59	116.14
N-nervonoyl valine	464.75	116.14
N-linoleoyl valine	378.58	116.14
N-docosahexaenoyl valine	426.62	116.14

**Free Fatty Acids**	**[M – H]$^-$**	**Fragment**
Linoleic Acid	279.5	261
Arachidonic Acid	303.5	285

**2-acyl-*sn*-glycerol**	**[M – H]$^-$**	**Fragment**
2-arachidonoyl-*sn*-glycerol	379.3	287.5
2-linoleoyl-*sn*-glycerol	355.5	245
2-oleoyl-*sn*-glycerol	357.5	265.2

**Prostaglandins**	**[M – H]$^-$**	**Fragment**
PGE_2	351.2	315
$PGF_{2\alpha}$	353.3	309.2

**Prostaglandin glycerol esters**	**[M – H]$^-$**	**Fragment**
PGE_2-G	444.5	391.2

Supplemental Table 1 List of lipids screened in HPLC/MS/MS analysis of NAPE-PLD KO, FAAH KO and WT mouse eyes with parent ion and fragment ion masses. Lipids are grouped by amide family; with all members screened in a multiple reactions monitoring (MRM) method. Negative ionization mode, resulting in a [M – H]$^-$ parent ion, is used for all methods except the *N*-acyl ethanolamine and 2-acyl glycerol methods, which uses positive ionization and generates a parent ion with a mass of [M + H]$^+$. The parent ion is then fragmented into the

collision chamber and an abundant fragment can be selected as the fragment ion. Therefore, unknown lipids are matched to known standards according to retention time from the analytical column and according to their mass fingerprint.
