# Supplementary material for: Evidence for a GPR18 Role in Diurnal Regulation of Intraocular Pressure
Source: Invest Ophthalmol Vis Sci. 2016 Nov;57(14):6419–26. doi: 10.1167/iovs.16-19437 (PMC5132083; doi:10.1167/iovs.16-19437)
Supplement: Supplement 2 [file iovs-57-13-48_s02.pdf]

SUPPLEMENTAL METHODS

Lipid Extraction

Six mice were used for each condition and were allowed at least two weeks to acclimatize to light condition. Control mice for a given condition (e.g. WT controls for FAAH and NAPE-PLD diurnal variation experiments, Fig. 2) were prepared at the same time and the samples analyzed together. Mice were sacrificed via cervical dislocation and both eyes were immediately removed and placed in an Eppendorf tube on dry ice. The eyes were then stored at -80 °C. To begin the lipid extraction, samples were shock frozen in liquid nitrogen, which allowed them to be easily removed from the Eppendorf tube and weighed before being transferred to a 15 mL centrifuge tube. The mass of the largest sample was multiplied by 50 to determine how many milliliters of HPLC-grade methanol (Avantor Performance Materials, Inc., Center Valley PA) was to be added to the centrifuge tube. Then, 5 µL of vortexed 1 µM deuterium-labeled *N*-arachidonoyl glycine (d_8NAGly) (Cayman Chemical, Ann Arbor, MI) was added to each test tube to serve as an internal standard. The spiked tubes were covered with Parafilm and were allowed to sit in the covered ice bucket for 2 hours. The eyes were then briefly homogenized using a sonicator (VirTis, Gardiner NY). Then, samples were spun in a centrifuge at 19,000g for 20 minutes at 20°C.

After centrifugation, supernatant was poured from the centrifuge tubes into 15 mL polypropylene tubes. Enough HPLC H_2O (EMD Millipore Corporation, Billerica, MA) to make a 75:25 water to organic solution was added to the supernatant. To partially purify the supernatant/water solution, solid phase extraction columns were used. One solid-phase 500mg C18 extraction cartridge (Agilent Technologies, Lake Forest CA) for each tube of extract was inserted into a Preppy vacuum manifold apparatus located in a fume hood. To activate the hydrophobic carbon chains in the column, 5 mL of HPLC methanol was added to each column. When the methanol almost reached the bottom of the columns, 2.5 mL of HPLC H_2O was added to the columns to activate the polar silica in the columns. When the water had almost run through the column, the supernatant/water solution was added and allowed to slowly drip through the column. After the solution had eluted, another 2.5 mL of HPLC H_2O was added to the columns to wash off impurities. Then, 1.5 mL of 40% methanol was added to the column to wash off more impurities. The 40% methanol was allowed to completely elute and any eluate in the collector vials was discarded. The collector vials were then replaced with labeled autosampler vials (Perkin Elmer, Waltham, MA) that corresponded to each sample. A series of 4 elutions with 1.5 mL of 60%, 75%, 85%, and 100% methanol as the eluting solvent was performed to partially purify the lipids being measured. More polar lipids, such as PGE_2 or $PGF_{2\alpha}$, were purified in the 60% and

75% elutions. On the other hand, lipids such as 2-AG and AEA were purified in the 100% elution and lipids such as NAGly were purified in the 85% elution. Vials of eluants were stored in the -80°C freezer until they were ready for analysis.

HPLC/MS/MS

80 lipids were chosen based on their structural relationship to canonical endocannabinoids AEA and 2-AG. Families of lipids include congeners that differ by the length and saturation of their carbon backbone (e.g. palmitoyl-, linoleoyl-, oleoyl-, arachidonoyl-, stearoyl-, docosahexaenoyl-) and are divided into families based on conjugation to amino acid head groups (glycerol, ethanolamine, serine, etc). Functional lipidomics studies of these lipids have found that some of these are active at other GPCRs, PPAR receptors and TRP channels (e.g. [33, 34]) while others may have as yet undetermined physiological properties. Samples were analyzed using an Applied Biosystems API 3000 triple quadrupole mass spectrometer (Applied Biosystems Sciex, Framingham, MA) with electrospray ionization. Levels of each compound were determined by running each sample using a multiple reactions monitoring (MRM) method tailored for each amide family of compounds. Samples were loaded with an autosampler (Shimadzu, Kyoto, Japan), which injected 20μL from each vial into the chromatography system for each method run. To chromatograph the

samples, an XDB-C18 (Agilent Technologies, Lake Forest CA) reversed phase HPLC analytical column was used, which was kept at 40°C by a column oven (HP, Palo Alto, CA). Two different types of mobile phase were used. Mobile phase A consisted of 20%/80% (v/v) methanol / water and 1 mM ammonium acetate (Sigma, St. Louis, MO). Mobile phase B instead contained 100% methanol with 1 mM ammonium acetate. Every method run began with 0% mobile phase B, reached a state of 100% mobile phase B flowing at 0.2 mL / minute, and gradually returned to 0% mobile phase B. Before running batches of samples, the ionization source was allowed to reach its operating temperature of 500°C and every vial warmed to room temperature and was vortexed for approximately 30 seconds.

Analysis of the HPLC/MS/MS data was performed using Analyst software. Chromatograms were generated by determining the retention time of analytes with a [M-1] or [M+1] parent peak and a fragmentation peak corresponding to the programmed values. The retention time was then compared to the retention time of a standard for the suspected compound. If the retention times matched, then the concentration of the compound was determined by calculating the area under the curve for the unknown and comparing it to the calibration curve obtained from the standards. Extraction efficiency was calculated with the d_8-NAGly spiked recovery vial as a standard. To determine the significant changes in lipid levels due to genotype, concentrations in moles per

gram adjusted for percent recovery from the knockout animals were compared to wild-type concentrations using a one-way ANOVA. For analysis of diurnal variation of lipid levels, concentrations in moles per gram adjusted for percent recovery for each lipid measured from eyes harvested at noon were compared to concentrations for lipids measured in eyes harvested at midnight using a one-way ANOVA.
All statistical tests for lipid measurements were carried out using SPSS Statistics 20 (IBM, Armonk, NY) or Graphpad Prism. Statistical significance was defined as $p \leq .05$ and a trending effect was defined as $0.05 < p \leq 0.10$. Unless otherwise noted, values are shown +/- SEM.

Quantitative Polymerase Chain Reaction Analysis

Primers for selected components of the endocannabinoid system were designed using Primer-Blast (http://www.ncbi.nlm.nih.gov/tools/primer-blast) and the corresponding mouse gene. Primer sequences are as listed, GPR18: 5'–AGC CAG CCT GCT GTA AGA TG–3' and 5'–GCC ACT CGC CTT TTG CAT AG–3'; FAAH: 5'–AGC CTC TGT TTC CTC GGC TGG–3'and 5'–TGG GCA TCG TCC TCA GCG GT–3'; NAPE: 5'–GGG TTT CGA CTT CTC GCC GAG GG–3' and 5'–CCA GCC TCT CTC ACT CCA GCG T–3'.

Tissue samples were extracted and immediately stored at -80C. RNA was extracted using a Trizol reagent (Ambion, Austin, TX) and genomic DNA was removed with DNase (NEB, Bethesda, MD) following the manufacturer’s

instructions. RT-PCR was performed using a one-step, Sybr Green amplification process (PwrSybr, Applied Biosystems, Carlsbad, CA). Quantitative PCR was performed using an Eppendorf RealPlex2 Mastercycler thermocycler.

A primer for b-actin was used as an internal control for each experimental condition with the threshold cycle set within the linear range (10 fold above baseline). Once the standard critical threshold (Ct) was set, the relative expression levels for genes were determined. Data analysis and statistics were performed using Excel (Microsoft Corp., Redmond, WA) and Prism (GraphPad Software Inc., San Diego, CA) software. Values were compared using an unpaired t test.
